# Agitation Management in the Emergency Department with Physical Restraints: Where Do These Patients End Up?

**DOI:** 10.5811/westjem.59466

**Published:** 2023-05-05

**Authors:** Erin L. Simon, Courtney M. Smalley, McKinsey Muir, Caroline Mangira Mangira, Rylee Pence, Bhanu Wahi-Singh, Fernando Delgado, Baruch S. Fertel

**Affiliations:** * Cleveland Clinic Akron General, Department of Emergency Medicine, Akron, Ohio; † Cleveland Clinic Emergency Services Institute, Cleveland, Ohio; ‡ Cleveland Clinic, Patient Quality and Safety, Cleveland, Ohio

## Abstract

**Introduction:**

Agitation is frequently encountered in the emergency department (ED) and can range from psychomotor restlessness to overt aggression and violent behavior. Among all ED patients, 2.6% present with agitation or become agitated during their ED visit. We aimed to determine ED disposition for patients requiring agitation management with physical restraints.

**Methods:**

This was a retrospective cohort of all adult patients who presented to one of 19 EDs in a large integrated healthcare system and received agitation management with physical restraints between January 1, 2018–December 31, 2020. Categorical variables are presented as frequency and percentages, and continuous variables are presented as medians and interquartile range.

**Results:**

There were 3,539 patients who had agitation management with physical restraints included in this study. In total 2,076 (58.8%) were admitted to the hospital (95% CI [confidence interval] 0.572–0.605), and of those 81.4% were admitted to a primary medical floor and 18.6% were medically cleared and admitted to a psychiatric unit. Overall, 41.2% were able to be medically cleared and discharged from the ED. Mean age was 40.9 years, 2,140 were male (59.1%), 1,736 were White (50.3%), and 1,527 (43%) were Black. We found 26% had abnormal ethanol, (95% CI 0.245–0.274) and 54.6% had an abnormal toxicology screen (95% CI 0.529–0.562). A significant number were administered a benzodiazepine or antipsychotic in the ED (88.44%) (95% CI 0.874–0.895).

**Conclusion:**

The majority of patients who had agitation management with physical restraints were admitted to the hospital; of those patients, 81.4% were admitted to a primary medical floor and 18.6% were admitted to a psychiatric unit.

## INTRODUCTION

Among all emergency department (ED) patients, 2.6% present with agitation or become agitated during their ED visit.^1^ Agitation describes a broad group of behaviors characterized by excessive motor or verbal activity manifesting as irritability, uncooperativeness, psychomotor restlessness, aggression, and violent behavior.^2^,^3^ As behavioral complaints and agitation become increasingly common in the ED, emergency physicians are tasked with maintaining both the safety and care of the patient and the safety of the staff and healthcare team. It has been reported that up to 50% of healthcare workers have experienced violence in their careers. A survey of emergency clinicians found that 78% had experienced violence in the workplace in the previous year.^4^–^6^

In the ED, the cause of agitation can be due to substance use disorder (SUD), psychiatric illness, or underlying medical illness. Therefore, early efforts in the ED should include identifying and treating reversible causes. However, in many cases of behavioral disturbance, intervention is indicated to reduce the risk of serious harm to patients and ED staff. Initial interventions to treat agitation may include non-coercive approaches such as verbal de-escalation;^7^,^8^ however, these techniques may be unsuccessful, and pharmacological sedation or restraint use may be necessary.

Despite the rate at which violence occurs in the ED, there is no standardized approach for managing agitation or objective measures for when agitation management with physical restraints is appropriate.^9^,^10^ Although the use of physical restraints has declined over the past several decades, they are still commonly used in the acute setting, with studies suggesting their use in over half of all acutely agitated patients.^11^–^13^ Non-medical physical restraints have been associated with significant morbidity and mortality with documented complications including restraint asphyxia, blunt trauma, catecholaminergic surge, and sudden death.^14^,^15^ Additionally, pharmacological management in an acutely agitated patient with comorbid medical conditions or SUD increases the risk of adverse respiratory events.^16^

### Importance

Given the heightened regulatory scrutiny and potential adverse events, physical restraints should be approached carefully. Many studies have evaluated the use of physical restraints in specific populations and the risk factors leading to restraint use.^17^–^19^ Few studies have examined the disposition of agitated patients and whether those admitted went to a primary medical or psychiatric unit. For example, one prior study examined the use of restraints on elderly patients and found that all patients were admitted to the hospital.^20^ Another study evaluated the length of stay and disposition of restrained patients who received an ED psychiatric consultation and found that approximately 70% were admitted to the hospital or a psychiatric facility.^21^ To our knowledge, no study has evaluated the characteristics and disposition of agitated patients who require management with physical restraints in a large, integrated healthcare system.

### Goals of the Investigation

We sought to determine patient disposition when the management of agitation with physical restraints is used in the ED.

## METHODS

### Study Design and Setting

This was a retrospective cohort of all adult patients who required agitation management with physical restraints across 19 EDs in a large, integrated healthcare system. All the EDs are located in northeast Ohio, except for one in southeast Florida, and include academic, community, freestanding, and critical access settings. Our study timeframe was January 1, 2018–December 31, 2020. The institutional review board approved this study.

**Population Health Research Capsule**

What do we already know about this issue?

*While use of physical restraints has declined, they are still used in the acute setting, with studies suggesting use in >50% of all acutely agitated patients*.

What was the research question?

*We sought to determine patient disposition when agitation is managed with physical restraints in the emergency department*.

What was the major finding of the study?

*We found that 58.8% of patients were admitted to the hospital (95% CI 0.572–0.605), with 81.4% of these admitted to a medical floor and 18.6% to psychiatry. Almost half (41.2%) were discharged from the ED after medical/psychiatric care*.

How does this improve population health?

*Consider a workup to assess for underlying medical conditions in patients requiring physical restraints and be cautious of anchoring on substance use as the cause of agitation*.

Whenever there is an escalating, potentially violent patient or situation in our healthcare system, caregivers may activate a “code violet.” Our healthcare system defines a “code violet” as a “violent or combative patient.” Once a “code violet” is initiated, an overhead page is sent out via the hospital-wide intercom system. This assembles a team with a Non-Abusive Psychological and Physical Intervention-trained team leader. This key communicator, who is typically the patient’s nurse, a hospital security officer, or a hospital police officer, first attempts to verbally de-escalate the patient in which no physical touch is used. If de-escalation fails, using reasonable physical force to protect caregivers, patients, and visitors from injury may be used. Next, pharmacological management or management with physical restraints may be used if deemed necessary to protect the safety of caregivers, patients, or visitors.

### Selection of Participants

Patients were included if they were ≥18 years of age and presented to one of 19 EDs within the healthcare system. In addition, to be included in the study participants must have had agitation management with physical restraints during their ED encounter.

### Data Collection

Study data were abstracted from the electronic health record (EHR) (Epic Systems Corporation, Verona, WI) via an automated query performed by one data analyst for the healthcare system. The data analyst was blinded to the study hypothesis. All patients who had management with physical restraints in the ED were required to have an order within the EHR. This was routinely audited by nursing and hospital quality leadership. We used Epic’s Clarity internal data warehouse to identify any ED encounters for which the “Restraint for Violent or Self-Destructive Behavior Management” order was applied. For all ED encounters with the “Violent Restraint” order, the original query was then expanded to collect additional data from the EHR. This included demographics, primary diagnosis of mental health condition, duration of restraint use, ED medications administered, ED lab toxicology screen results, ED medical clearance, ED disposition, and whether an ED psychiatric intake encounter occurred.

### Outcomes

The primary outcome was ED disposition for patients requiring agitation management with physical restraints. Secondary outcomes included duration of physical restraint use, use of pharmacological management (antipsychotics or benzodiazepines) in the ED, primary diagnosis of behavioral health disorder in the ED, history of dementia, abnormal ED ethanol results, abnormal ED toxicology results, ED length of stay (LOS), and whether an ED psychiatric intake encounter occurred. Behavioral health disorders were determined using the International Classification of Diseases, 10^th^ Ed, codes from the “Meaningful Use” recommendation and are included in the appendix.

### Statistical Analysis

Categorical variables are presented as frequency and percentages, and continuous variables are presented as medians and interquartile ranges (IQR).

## RESULTS

There were 3,539 patients who had management with physical restraints during the study timeframe. Overall, the mean age was 40.9 years; 2,140 59.1% were male (59.1%), 1,736 (49.1%) were White, and 1,522 (43%) were Black. Overall, 22.2% had Medicare, 53.7% had Medicaid, 12.3% had private insurance, and 10.7% were self-pay (Table 1).

For our primary outcome, we found that 2,076 patients (58.7%) were admitted to the hospital (95% confidence interval [CI] 0.572–0.605). Of those patients, 1,172 (56.5%) were admitted to a primary medical unit, 518 (25.0%) were admitted to an intensive care unit, and 386 (18.6%) were medically cleared and admitted to a psychiatric unit. Three patients expired in the ED, two of them due to critical illness and unrelated to restraint use. One death was due to cardiac arrest of unclear etiology while in restraints. Overall, 41.2% were medically cleared and discharged from the ED. Table 2 shows characteristics of patients based on ED disposition (admitted vs discharged).

We also found that most patients had a primary mental health diagnosis (54.5%) in the ED, while only 7.9% had a prior history of dementia. We found that 29.6% had elevated ethanol levels (≥11 milligrams per deciliter), and 59.7% had an abnormal toxicology screen. A significant number were administered a benzodiazepine (80.2%) or antipsychotic (71.2%) while in the ED, and 42.2% had an evaluation by the ED psychiatric intake team (Table 2).

The overall median ED LOS was 495 minutes; ED LOS was 463 minutes for admitted patients and 526 minutes for discharged patients. Overall median minutes in restraints was 99 minutes: 98 minutes for admitted patients and 100 minutes for discharged patients (Table 3). We then tested the association of the characteristics from Table 3 in Table 4.

## DISCUSSION

There is limited information characterizing agitated patients managed with physical restraints in the ED and their disposition. Agitation can be multifactorial, and its causes include underlying medical issues, SUD, psychotic episodes, and non-psychotic psychiatric illness. Understanding this patient population allows for more informed use of restraints in the ED.^17^

When evaluating the primary outcome of our study, the ED disposition of patients managed with physical restraints varied. Only 18.6% of admitted patients were admitted to a psychiatric unit, showing that agitation is a multifactorial process and is often not solely psychiatric. Additionally, 41% of patients managed with physical restraints were successfully de-escalated, medically cleared, and discharged home after ED evaluation. This supports prior literature that management with physical restraints is often temporary, and many patients can be discharged from the ED.^21^

Patients who are agitated are often assumed to be under the influence of a behavior-modifying substance. This can lead clinicians to chemically sedate and physically restrain them for safety, allowing time for the behavior-modifying substance to wear off, with no additional workup being undertaken. We found that of patients admitted, 81.4% required admission to a primary medical unit for an underlying medical condition. Just under half of all patients evaluated did not have a primary mental health diagnosis in the ED. These numbers help illustrate that a substantial portion of agitated patients managed with physical restraints do not have a psychiatric etiology for their agitation, and a medical workup for other causes should be undertaken.

Our secondary outcomes help to define characteristics of agitated patients managed with physical restraints. Substance use was present in a substantial number of those restrained, with many having either abnormal ethanol levels or an abnormal toxicology screen. The ED often manages and observes patients whose agitation is thought to be primarily due to an underlying SUD. Emergency clinicians typically wait until the substance has cleared from a patient’s system and the patient has the capacity to make decisions, which may explain why patients discharged had a longer ED LOS. We found that time in restraints was similar whether patients were admitted or discharged regardless of the underlying cause. This may be due to an overall goal of our healthcare system to minimize the time agitated patients are managed with physical restraints. Despite pharmacological management for agitation being used in most cases, we found physical restraints were additionally needed to maintain safety in the ED setting. We found that the frequency with which pharmacological management occurred was similar for admitted or discharged patients. Pharmacological management of agitation is done with the goal to help to calm the patient. It can be a valuable adjunct to management with physical restraints in maintaining safety while determining how to evaluate best and manage the underlying cause.^18^

## LIMITATIONS

This study has several limitations. First, this was a retrospective study based on EHR data; therefore, it depends on proper documentation for the data points assessed. It is unlikely that restraint use was not documented, or that patients were missed in our cohort. A physical restraint order is required for restraints to be placed on all violent or agitated patients throughout the healthcare system. Also, our population represents data from a single, large, integrated healthcare system. Since the healthcare system is primarily located in one region of the United States, our results may not be generalizable to other regions. Finally, we could not differentiate whether a patient who was positive on the toxicology screen for benzodiazepines had taken them prior to arrival or whether they were administered in the ED for agitation.

## CONCLUSION

Most patients with agitation management with physical restraints were admitted to the hospital to a primary medical floor due to an underlying medical condition. This emphasizes the importance of a thorough workup to assess for underlying medical conditions and to be cautious of anchoring on substance use disorder as the cause of their agitation.

## Supplementary Information



## Figures and Tables

**Table 1:** Demographics of patients in study of use of physical restraints in the emergency department.

Demographics	ED encounters	Admitted	Discharged
Gender			
Male	2,093 (59.1%)	1,142 (32.3%)	951 (26.9%)
Female	1,445 (40.8%)	948 (26.8%)	497 (14.0%)
Unknown	1	1 (0%)	0
Total	3,539	2,091 (59.1%)	1,448 (40.9%)
Insurance			
Medicaid HMO	1,901 (53.7%)	1,041(29.4%)	860 (24.3%)
Medicare HMO	784 (22.2%)	597 (16.9%)	187 (5.3%)
Self-pay	381 (10.8%)	154 (4.4%)	227 (6.4%)
Private	437 (12.3%)	280 (7.9%)	157 (4.4%)
Left blank	36 (1.0%)	19 (0.5%)	17 (0.5%)
Total	3,539	2,091 (59.1%)	1,448 (40.9%)
Race			
White	1,736 (49.1%)	1,130 (31.9%)	606 (17.1%)
Black	1,522 (43.0%)	790 (22.3%)	732 (20.7%)
Multiracial	93 (2.6%)	51 (1.4%)	42 (1.2%)
Other	178 (5.0%)	112 (3.2%)	66 (1.9%)
Asian	7 (0.2%)	5 (0.1%)	2 (0.1%)
American Indian/Alaskan Native	3 (0.1%)	3 (0.1%)	0
Grand total	3,539	2,091 (59.1%)	1,448 (40.9%)

*ED*, emergency department; *HMO*, health maintenance organization.

**Table 2:** Characteristics of participants based on whether they were admitted or discharged, N=3,539.

Variable	Overall(N=3,539)		Admitted (n=2,076)		Discharged (n=1,452)	
		
	N (column %)	95% CI	n (column %)	95% CI	n (column %)	95% CI
Primary diagnosis of behavioral health	1,928 (54)	53 – 56	847 (44.14)	39 – 43	1,072 (74)	72 – 76
Dementia present in the problem list	278 (8)	7 – 9	248 (12)	11 – 13	30 (2)	1 – 3
Psychiatry intake encounter	1,493 (42)	41 – 44	1,028 (50)	47 – 52	455(31)	29 – 33
Antipsychotics administered in ED	2,521 (71)	70 – 73	1,461(70)	68 – 72	1,050 (72)	70 – 75
Benzodiazepines administered in ED	2,838 (80)	79 – 82	1,687(81)	80 – 83	1,141(79)	76 – 81
Both benzodiazepines and antipsychotics administered in ED	2,230 (63)	61 – 65	1,306 (63)	61 – 65	915 (63)	61 – 66
Results positive for opioids	162 (5)	4 – 6	110 (5)	4 – 6	52 (4)	3 – 5
Results positive for benzodiazepines	268 (8)	7 – 9	192 (9)	8 – 10	76 (5)	4 – 6
Results positive for ethanol	1,046 (30)	28 – 31	368(18)	16 – 19	676(47)	43 – 49
Positive toxicology screen	2,113 (60)	58 – 61	1,127 (54)	52 – 56	982 (68)	65 – 70
ED chief complaint of suicidal ideation or suicide attempt	370 (10)	9 – 11	236 (11)	10 – 13	133 (9)	8 – 11
ED chief complaint of ethanol problem	378 (11)	10 – 12	103 (5)	4 – 6	275 (19)	17 – 21
ED chief complaint of Intoxication	295 (8)	7 – 9	53 (3)	2 – 3	242 (16.67)	15 – 19
	Median (IQR)	95% CI	Median (IQR)	95% CI	Median (IQR)	95% CI
ED LOS (min.)	495 (443)	478 – 509	463 (529)	441 – 478	526 (349)	511 – 539
Total minutes in restraints	99 (150)	94 – 105	98 (170)	90 – 105	100 (129)	91 – 105

*ED*, emergency department; *LOS*, length of stay; *CI*, confidence interval; *IQR*, interquartile range.

**Table 3:** Characteristics of participants based on disposition [for results (+) benzodiazepines, unable to determine whether taken prior to emergency department (ED) visit or given in the ED].

Variable	Overall		Admitted		Discharged	
		
	N (%) or Median (IQR)	95% CI	N (%) or Median (IQR)	95% CI	N (%) or Median (IQR)	95% CI
ED LOS (min.)	495 (443)	478 – 509	463 (529)	441 – 478	526 (349)	511 – 539
Total minutes in restraints	99 (150)	94 – 105	98 (170)	90 – 105	100 (129)	91 – 105
Psychiatry consults	1,493 (42%)	41 – 44	1,028 (50%)	47 – 52	455 (31%)	29 – 33
Results (+) for opioids	162 (5%)	4 – 6	110 (5%)	4 – 6	52 (4%)	3 – 5
Results (+) for benzodiazepines	268 (8%)	7 – 9	192 (9%)	8 – 10	76 (5%)	4 – 6
Results (+) for ethanol	1,046 (30%)	28 – 31	368(18%)	16 – 19	676 (47%)	43 – 49
Medicated with benzodiazepines	2,838 (80%)	79 – 82	1,687 (81%)	80 – 83	1,141 (79%)	76 – 81
Medicated with antipsychotics	2521 (71%)	70 – 73	1,461 (70%)	68 – 72	1,050 (72%)	70 – 75

*ED*, emergency department; *IQR*, interquartile range; *CI*, confidence interval; *LOS*, length of stay.

**Table 4:** Associations of different characteristics by admission status (admitted vs discharged).

Variable	Admitted	Discharged	IM or RD (95% CI)	P-value
ED LOS (minutes)	463 (529)	526 (349)	−19.00 (−39.00, 2.00)	0.081
Total minutes with restraints	98 (170)	100 (129)	1.00 (−5.00, 6.00)	0.860
Psychiatry consults				
Yes	1,028 (69.32)	455 (30.68)	18.07 (14.88, 21.27)	<0.0001
No	1,048(51.25)	997 (48.75)		
Results positive for opioids				
Yes	110 (67.90)	52 (32.10)	7.91 (0.49, 15.32)	0.045
No	1,669 (59.99)	1,113 (40.01)		
Results positive for benzodiazepines				
Yes	192 (71.64)	76 (28.36)	12.34 (6.63, 18.05)	<0.0001
No	1,587 (59.30)	1,089 (40.70)		
Results positive for ethanol				
Yes	368 (35.25)	676 (64.75)	−39.01 (−42.52, −35.51)	<0.0001
No	1,411 (74.26)	489 (25.74)		
Positive toxicology screen				
Yes	1,127 (53.44)	982 (46.56)	−13.44(−16.69, −10.20)	<0.0001
No	949 (66.88)	470 (33.12)		
Used benzodiazepines				
Yes	1,687 (59.65)	1,141 (40.35)	4.08 (−0.02, 8.18)	0.054
No	389(55.57)	311 (44.43)		
Used antipsychotics				
Yes	1,461 (58.18)	1,050 (41.82)	−2.29 (−5.86, 1.28)	0.211
No	615 (60.47)	402 (39.53)		

Results presented as median (IQR) or n (row %).

*IM*, interval midpoint; *RD*, risk difference; *CI*, confidence interval; *ED*, emergency department; *LOS*, length of stay.
