# Supplementary material for: Agitation Management in the Emergency Department with Physical Restraints: Where Do These Patients End Up?
Source: West J Emerg Med. 2023 May 5;24(3):454–60. doi: 10.5811/westjem.59466 (PMC10284532; doi:10.5811/westjem.59466)
Supplement: Supplementary file 1 [file wjem-24-454-Appendix.docx]

Appendix.

Behavioral Health ICD-10 Codes:

| **ICD_10_Code** |
| --- |
| F01.50 |
| F01.51 |
| F03.90 |
| F03.91 |
| F04 |
| F05 |
| F06.0 |
| F06.1 |
| F06.2 |
| F06.30 |
| F06.31 |
| F06.32 |
| F06.34 |
| F06.4 |
| F06.8 |
| F07.0 |
| F07.81 |
| F07.89 |
| F07.9 |
| F09 |
| F10.10 |
| F10.120 |
| F10.121 |
| F10.129 |
| F10.14 |
| F10.180 |
| F10.188 |
| F10.19 |
| F10.20 |
| F10.21 |
| F10.220 |
| F10.221 |
| F10.229 |
| F10.230 |
| F10.231 |
| F10.232 |
| F10.239 |
| F10.24 |
| F10.251 |
| F10.259 |
| F10.26 |
| F10.27 |
| F10.280 |
| F10.281 |
| F10.282 |
| F10.288 |
| F10.29 |
| F10.920 |
| F10.921 |
| F10.929 |
| F10.94 |
| F10.950 |
| F10.951 |
| F10.959 |
| F10.96 |
| F10.97 |
| F10.980 |
| F10.981 |
| F10.982 |
| F10.988 |
| F10.99 |
| F11.10 |
| F11.120 |
| F11.121 |
| F11.122 |
| F11.129 |
| F11.14 |
| F11.150 |
| F11.151 |
| F11.159 |
| F11.181 |
| F11.182 |
| F11.188 |
| F11.19 |
| F11.20 |
| F11.21 |
| F11.220 |
| F11.221 |
| F11.222 |
| F11.229 |
| F11.23 |
| F11.24 |
| F11.250 |
| F11.251 |
| F11.259 |
| F11.281 |
| F11.282 |
| F11.288 |
| F11.29 |
| F11.90 |
| F11.920 |
| F11.921 |
| F11.922 |
| F11.929 |
| F11.93 |
| F11.94 |
| F11.950 |
| F11.951 |
| F11.959 |
| F11.981 |
| F11.982 |
| F11.988 |
| F11.99 |
| F12.10 |
| F12.120 |
| F12.121 |
| F12.122 |
| F12.129 |
| F12.150 |
| F12.151 |
| F12.159 |
| F12.180 |
| F12.188 |
| F12.19 |
| F12.20 |
| F12.21 |
| F12.220 |
| F12.221 |
| F12.222 |
| F12.229 |
| F12.250 |
| F12.251 |
| F12.259 |
| F12.280 |
| F12.288 |
| F12.29 |
| F12.90 |
| F12.920 |
| F12.921 |
| F12.922 |
| F12.929 |
| F12.950 |
| F12.951 |
| F12.959 |
| F12.980 |
| F12.988 |
| F12.99 |
| F13.10 |
| F13.120 |
| F13.121 |
| F13.129 |
| F13.14 |
| F13.150 |
| F13.151 |
| F13.159 |
| F13.180 |
| F13.181 |
| F13.182 |
| F13.188 |
| F13.19 |
| F13.20 |
| F13.21 |
| F13.220 |
| F13.221 |
| F13.229 |
| F13.230 |
| F13.231 |
| F13.232 |
| F13.239 |
| F13.24 |
| F13.250 |
| F13.251 |
| F13.259 |
| F13.26 |
| F13.27 |
| F13.280 |
| F13.281 |
| F13.282 |
| F13.288 |
| F13.29 |
| F13.90 |
| F13.920 |
| F13.921 |
| F13.929 |
| F13.930 |
| F13.931 |
| F13.932 |
| F13.939 |
| F13.94 |
| F13.950 |
| F13.951 |
| F13.959 |
| F13.96 |
| F13.97 |
| F13.980 |
| F13.981 |
| F13.982 |
| F13.988 |
| F13.99 |
| F14.10 |
| F14.120 |
| F14.121 |
| F14.122 |
| F14.129 |
| F14.14 |
| F14.150 |
| F14.151 |
| F14.159 |
| F14.180 |
| F14.181 |
| F14.182 |
| F14.188 |
| F14.19 |
| F14.20 |
| F14.21 |
| F14.220 |
| F14.221 |
| F14.222 |
| F14.229 |
| F14.23 |
| F14.24 |
| F14.250 |
| F14.251 |
| F14.259 |
| F14.280 |
| F14.281 |
| F14.282 |
| F14.288 |
| F14.29 |
| F14.90 |
| F14.920 |
| F14.921 |
| F14.922 |
| F14.929 |
| F14.94 |
| F14.950 |
| F14.951 |
| F14.959 |
| F14.980 |
| F14.981 |
| F14.982 |
| F14.988 |
| F14.99 |
| F15.10 |
| F15.120 |
| F15.121 |
| F15.122 |
| F15.129 |
| F15.14 |
| F15.150 |
| F15.151 |
| F15.159 |
| F15.180 |
| F15.181 |
| F15.182 |
| F15.188 |
| F15.19 |
| F15.20 |
| F15.21 |
| F15.220 |
| F15.221 |
| F15.222 |
| F15.229 |
| F15.23 |
| F15.24 |
| F15.250 |
| F15.251 |
| F15.259 |
| F15.280 |
| F15.281 |
| F15.282 |
| F15.288 |
| F15.29 |
| F15.90 |
| F15.920 |
| F15.921 |
| F15.922 |
| F15.929 |
| F15.93 |
| F15.94 |
| F15.950 |
| F15.951 |
| F15.959 |
| F15.980 |
| F15.981 |
| F15.982 |
| F15.988 |
| F15.99 |
| F16.10 |
| F16.120 |
| F16.121 |
| F16.122 |
| F16.129 |
| F16.14 |
| F16.150 |
| F16.151 |
| F16.159 |
| F16.180 |
| F16.183 |
| F16.188 |
| F16.19 |
| F16.20 |
| F16.21 |
| F16.220 |
| F16.221 |
| F16.229 |
| F16.24 |
| F16.250 |
| F16.251 |
| F16.259 |
| F16.280 |
| F16.283 |
| F16.288 |
| F16.29 |
| F16.90 |
| F16.920 |
| F16.921 |
| F16.929 |
| F16.94 |
| F16.950 |
| F16.951 |
| F16.959 |
| F16.980 |
| F16.983 |
| F16.988 |
| F16.99 |
| F18.10 |
| F18.120 |
| F18.121 |
| F18.129 |
| F18.14 |
| F18.150 |
| F18.151 |
| F18.159 |
| F18.17 |
| F18.180 |
| F18.188 |
| F18.19 |
| F18.20 |
| F18.21 |
| F18.220 |
| F18.221 |
| F18.229 |
| F18.24 |
| F18.250 |
| F18.251 |
| F18.259 |
| F18.27 |
| F18.280 |
| F18.288 |
| F18.29 |
| F18.90 |
| F18.920 |
| F18.921 |
| F18.929 |
| F18.94 |
| F18.950 |
| F18.951 |
| F18.959 |
| F18.97 |
| F18.980 |
| F18.988 |
| F18.99 |
| F19.10 |
| F19.120 |
| F19.121 |
| F19.122 |
| F19.129 |
| F19.14 |
| F19.150 |
| F19.151 |
| F19.159 |
| F19.16 |
| F19.17 |
| F19.180 |
| F19.181 |
| F19.182 |
| F19.188 |
| F19.19 |
| F19.20 |
| F19.21 |
| F19.220 |
| F19.221 |
| F19.222 |
| F19.229 |
| F19.230 |
| F19.231 |
| F19.232 |
| F19.239 |
| F19.24 |
| F19.250 |
| F19.251 |
| F19.259 |
| F19.26 |
| F19.27 |
| F19.280 |
| F19.281 |
| F19.282 |
| F19.288 |
| F19.29 |
| F19.90 |
| F19.920 |
| F19.921 |
| F19.922 |
| F19.929 |
| F19.930 |
| F19.931 |
| F19.932 |
| F19.939 |
| F19.94 |
| F19.950 |
| F19.951 |
| F19.959 |
| F19.96 |
| F19.97 |
| F19.980 |
| F19.981 |
| F19.982 |
| F19.988 |
| F19.99 |
| F20.0 |
| F20.1 |
| F20.2 |
| F20.3 |
| F20.5 |
| F20.81 |
| F20.89 |
| F20.9 |
| F21 |
| F22 |
| F23 |
| F24 |
| F25.0 |
| F25.1 |
| F25.8 |
| F25.9 |
| F28 |
| F29 |
| F30.10 |
| F30.11 |
| F30.12 |
| F30.13 |
| F30.2 |
| F30.3 |
| F30.4 |
| F30.8 |
| F30.9 |
| F31.0 |
| F31.10 |
| F31.11 |
| F31.12 |
| F31.13 |
| F31.2 |
| F31.30 |
| F31.31 |
| F31.32 |
| F31.4 |
| F31.5 |
| F31.60 |
| F31.61 |
| F31.62 |
| F31.63 |
| F31.64 |
| F31.70 |
| F31.71 |
| F31.72 |
| F31.73 |
| F31.74 |
| F31.75 |
| F31.76 |
| F31.77 |
| F31.78 |
| F31.81 |
| F31.89 |
| F31.9 |
| F32.0 |
| F32.1 |
| F32.2 |
| F32.3 |
| F32.4 |
| F32.5 |
| F32.81 |
| F32.89 |
| F32.9 |
| F33.0 |
| F33.1 |
| F33.2 |
| F33.3 |
| F33.40 |
| F33.41 |
| F33.42 |
| F33.8 |
| F33.9 |
| F34.0 |
| F34.1 |
| F34.81 |
| F34.89 |
| F34.9 |
| F39 |
| F40.00 |
| F40.01 |
| F40.02 |
| F40.10 |
| F40.11 |
| F40.210 |
| F40.218 |
| F40.220 |
| F40.228 |
| F40.230 |
| F40.231 |
| F40.232 |
| F40.233 |
| F40.240 |
| F40.241 |
| F40.242 |
| F40.243 |
| F40.248 |
| F40.290 |
| F40.291 |
| F40.298 |
| F40.8 |
| F40.9 |
| F41.0 |
| F41.1 |
| F41.3 |
| F41.8 |
| F41.9 |
| F42.2 |
| F42.3 |
| F42.4 |
| F42.8 |
| F42.9 |
| F43.0 |
| F43.10 |
| F43.11 |
| F43.12 |
| F43.20 |
| F43.21 |
| F43.22 |
| F43.23 |
| F43.24 |
| F43.25 |
| F43.29 |
| F43.8 |
| F43.9 |
| F44.0 |
| F44.1 |
| F44.2 |
| F44.4 |
| F44.5 |
| F44.6 |
| F44.7 |
| F44.81 |
| F44.89 |
| F44.9 |
| F45.0 |
| F45.1 |
| F45.20 |
| F45.21 |
| F45.22 |
| F45.29 |
| F45.41 |
| F45.42 |
| F45.8 |
| F45.9 |
| F48.1 |
| F48.2 |
| F48.8 |
| F48.9 |
| F50.00 |
| F50.01 |
| F50.02 |
| F50.2 |
| F50.81 |
| F50.89 |
| F50.9 |
| F51.01 |
| F51.02 |
| F51.03 |
| F51.04 |
| F51.05 |
| F51.09 |
| F51.11 |
| F51.12 |
| F51.13 |
| F51.19 |
| F51.3 |
| F51.4 |
| F51.5 |
| F51.8 |
| F51.9 |
| F52.0 |
| F52.1 |
| F52.21 |
| F52.22 |
| F52.31 |
| F52.32 |
| F52.4 |
| F52.5 |
| F52.6 |
| F52.8 |
| F52.9 |
| F53 |
| F55.0 |
| F55.1 |
| F55.2 |
| F55.3 |
| F55.4 |
| F55.8 |
| F59 |
| F60.0 |
| F60.1 |
| F60.2 |
| F60.3 |
| F60.4 |
| F60.5 |
| F60.6 |
| F60.7 |
| F60.81 |
| F60.89 |
| F60.9 |
| F63.0 |
| F63.1 |
| F63.2 |
| F63.3 |
| F63.81 |
| F63.89 |
| F63.9 |
| F64.0 |
| F64.1 |
| F64.2 |
| F64.8 |
| F64.9 |
| F65.0 |
| F65.1 |
| F65.2 |
| F65.3 |
| F65.4 |
| F65.50 |
| F65.51 |
| F65.52 |
| F65.81 |
| F65.89 |
| F65.9 |
| F66 |
| F68.10 |
| F68.11 |
| F68.12 |
| F68.13 |
| F68.8 |
| F69 |
| F70 |
| F71 |
| F72 |
| F73 |
| F78 |
| F79 |
| F80.0 |
| F80.1 |
| F80.2 |
| F80.4 |
| F80.81 |
| F80.82 |
| F80.89 |
| F80.9 |
| F81.0 |
| F81.2 |
| F81.81 |
| F81.89 |
| F81.9 |
| F82 |
| F84.0 |
| F84.2 |
| F84.3 |
| F84.5 |
| F84.8 |
| F84.9 |
| F88 |
| F89 |
| F90.0 |
| F90.1 |
| F90.2 |
| F90.8 |
| F90.9 |
| F91.0 |
| F91.1 |
| F91.2 |
| F91.3 |
| F91.8 |
| F91.9 |
| F93.0 |
| F93.8 |
| F93.9 |
| F94.0 |
| F94.1 |
| F94.2 |
| F94.8 |
| F94.9 |
| F95.0 |
| F95.1 |
| F95.2 |
| F95.8 |
| F95.9 |
| F98.0 |
| F98.1 |
| F98.21 |
| F98.29 |
| F98.3 |
| F98.4 |
| F98.5 |
| F98.8 |
| F98.9 |
| F99 |
| G25.61 |
| R37 |
| R40.1 |
| R40.4 |
| R41.0 |
| R41.1 |
| R41.2 |
| R41.3 |
| R41.82 |
| R41.89 |
| R44.0 |
| R44.1 |
| R44.2 |
| R44.3 |
| R44.8 |
| R45.0 |
| R45.1 |
| R45.2 |
| R45.3 |
| R45.4 |
| R45.5 |
| R45.6 |
| R45.7 |
| R45.81 |
| R45.82 |
| R45.83 |
| R45.84 |
| R45.850 |
| R45.851 |
| R45.87 |
| R45.89 |
| R46.1 |
| R46.2 |
| R46.3 |
| R46.5 |
| R46.6 |
| R46.7 |
| R46.81 |
| R46.89 |
| Z55.4 |
| Z56.3 |
| Z56.4 |
| Z56.81 |
| Z59.2 |
| Z60.0 |
| Z60.3 |
| Z60.4 |
| Z60.5 |
| Z62.0 |
| Z62.1 |
| Z62.22 |
| Z62.29 |
| Z62.3 |
| Z62.6 |
| Z62.810 |
| Z62.811 |
| Z62.812 |
| Z62.819 |
| Z62.820 |
| Z62.821 |
| Z62.822 |
| Z62.890 |
| Z62.891 |
| Z62.898 |
| Z62.9 |
| Z63.0 |
| Z63.1 |
| Z63.4 |
| Z63.5 |
| Z63.71 |
| Z63.72 |
| Z63.79 |
| Z63.8 |
| Z64.4 |
| Z65.8 |
| Z69.010 |
| Z69.011 |
| Z69.020 |
| Z69.021 |
| Z69.11 |
| Z69.12 |
| Z69.81 |
| Z69.82 |
| Z70.0 |
| Z70.1 |
| Z70.2 |
| Z70.3 |
| Z70.8 |
| Z70.9 |
| Z71.41 |
| Z71.42 |
| Z71.51 |
| Z71.52 |
| Z71.89 |
| Z72.51 |
| Z72.52 |
| Z72.53 |
| Z72.6 |
| Z72.810 |
| Z72.811 |
| Z72.820 |
| Z72.821 |
| Z72.89 |
| Z73.0 |
| Z73.1 |
| Z73.3 |
| Z73.4 |
| Z73.5 |
| Z73.810 |
| Z73.811 |
| Z73.812 |
| Z73.819 |
| Z73.89 |
| Z86.51 |
| Z86.59 |
| Z87.820 |
| Z87.890 |
| Z91.410 |
| Z91.411 |
| Z91.412 |
| Z91.419 |
| Z91.49 |
| Z91.5 |
